# Evaluation of dental pulp stem cells behavior after odontogenic differentiation induction by three different bioactive materials on two different scaffolds

**DOI:** 10.1186/s12903-023-02975-3

**Published:** 2023-05-01

**Authors:** Basma Ahmed, Mai H. Ragab, Rania A. Galhom, Hayam Y. Hassan

**Affiliations:** 1 Endodontic Department, Faculty of Dentistry, Suez Canal University, Ismailia, Egypt; 2 Human Anatomy and Embryology Department, Faculty of Medicine, Suez Canal University, Ismailia, Egypt

**Keywords:** Dental pulp stem cells, Activa bioactive, TeraCal LC, Mineral trioxide aggregate

## Abstract

**Background:**

To study the odontogenic potential of dental pulp stem cells (DPSCs) after induction with three different bioactive materials: activa bioactive (base/liner) (AB), TheraCal LC (TC), and mineral trioxide aggregate (MTA), when combined with two different types of scaffolds.

**Methods:**

DPSCs were isolated from freshly extracted premolars of young orthodontic patients, cultured, expanded to passage 4 (P), and characterized by flow cytometric analysis. DPSCs were seeded onto two scaffolds in contact with different materials (AB, TC, and MTA). The first scaffold contained polycaprolactone-nano-chitosan and synthetic hydroxyapatite (PCL-NC-HA), whereas the second scaffold contained polycaprolactone-nano-chitosan and synthetic Mg-substituted hydroxyapatite (PCL-NC-Mg-HA). DPSC viability and proliferation were evaluated at various time points. To assess odontoblastic differentiation, gene expression analysis of dentin sialophosphoprotein (DSPP) by quantitative real-time polymerase chain reaction (qRT-PCR) and morphological changes in cells were performed using inverted microscope phase contrast images and scanning electron microscopy. The fold-change in DSPP between subgroups was compared using a one-way ANOVA. Tukey's test was used to compare the fold-change in DSPP between the two subgroups in multiple comparisons, and P was set at *p* < 0.05.

**Results:**

DSPP expression was significantly higher in the PCL-NC-Mg-HA group than in the PCL-NC-HA group, and scanning electron microscopy revealed a strong attachment of odontoblast-like cells to the scaffold that had a stronger odontogenic differentiation effect on DPSCs than the scaffold that did not contain magnesium. MTA has a significantly higher odontogenic differentiation effect on cultured DPSCs than AB or TC does. The combination of scaffolds and bioactive materials improves DPSCs induction in odontoblast-like cells.

**Conclusions:**

The PCL-NC-Mg-HA scaffold showed better odontogenic differentiation effects on cultured DPSCs. Compared to AB and TC, MTA is the most effective bioactive material for inducing the odontogenic differentiation of cultured DPSCs.

## Introduction

Teeth loss due to pathological causes remains a reality in clinical dentistry and results in functional and psychological complications. Tissue engineering, which has been studied in dentistry for several years, is a new option for addressing this problem. This field is thought to be multidisciplinary [1].

Regenerative endodontics is based on the concept of tissue engineering, which comprises of three key elements: stem cells, scaffolds, and growth factors. Human exfoliated deciduous teeth (SHED) and dental pulp stem cells (DPSCs) from permanent teeth are multipotent, which means that they can differentiate into odontogenic, angiogenic, adipogenic, chondrogenic, neurogenic, or myogenic cells [2, 3].

Many studies have revealed that in vitro DPSCs have been shown to produce dense calcified nodules, and when recombined with biodegradable scaffolds, they can form irregularly shaped dentin pulp-like tissues [4–6].

Bioactive materials cause a specific biological response at the material interface, resulting in the formation of a bond between tissues and the material. Mineral trioxide aggregate (MTA) is a bioactive material that induces the differentiation and proliferation of stem cells, resulting in the formation of a dentinal bridge that thickens over time [7, 8].

Recently, commercially available glass-incorporated light-curable pulp-capping materials known as Activa bioactive (AB) and TheraCal LC (TC), which are hybrid bioactive materials, have been introduced as promising bioactive materials [9].

Many studies have been conducted in the last few years to investigate the odontogenic differentiation of DPSCs, both in vitro and in vivo, and DPSCs have been reported to be critical for reparative dentin formation and regeneration of the dentin–pulp complex. Some studies have used scaffolds, whereas others have used bioactive materials to achieve odontogenic differentiation of DPSCs [10].

The odontogenic differentiation of dental pulp stem cells can be measured using gene expression technology, specifically two-step quantitative real-time polymerase chain reaction (qRT-PCR), which has been proven to be the most reliable method for gene expression analysis in recent years. This is a quick and easy polymerase chain reaction (PCR) method that uses fluorogenic primers. Observers can monitor the PCR reaction by detecting changes in fluorescence intensity during the reaction [11].

The gene of choice for this study was dentin sialophosphoprotein (DSPP), which is primarily expressed in odontoblasts. DSPP expression is higher during primary dentinogenesis and plays a more aggressive role in odontoblast differentiation and function during primary dentinogenesis [12].

Few studies on the effect of bioactive materials combined with the effect of scaffolds on DPSCs have been published; thus, the current study aimed to investigate the odontogenic effect of glass-incorporated light-curable bioactive material AB in comparison with TC and MTA when combined with the effect of two different types of scaffolds. DSPP gene expression analysis by qRT-PCR was used to assess the odontogenic differentiation of cultured DPSCs. The null hypothesis suggested no difference would be reported between the used bioactive materials nor the used scaffold.

## Materials and methods

The manuscript of this laboratory study has been written according to the Preferred Reporting Items for Laboratory Studies in Endodontology (PRILE) 2021 guidelines [13]. This research was approved by the ethical committee of the Faculty of Dentistry, Suez Canal University (approval no. (191–2019), all methods were carried out in accordance with relevant guidelines and regulations.

### Cell isolation and culture

Written consent was obtained from 20 young patients, aged–18–25 years. The patients were scheduled for extraction of sound permanent premolars during orthodontic treatment. The eligibility criteria included teeth with mature apices with no resorption, calcifications, or any other periodontal diseases indicated for extraction; teeth with periodontal lesions; and carious partially impacted teeth.

Twenty sound permanent teeth were stored in sterile falcon tubes containing a storage medium composed of Dulbecco’s phosphate-buffered saline (Biowest, Canada) (DMEM) and 5% penicillin streptomycin (Sigma, USA). The teeth were then transferred to the tissue culture laboratory for pulp extirpation and stem cell isolation.

In a laminar flow hood (Thermo Fisher Scientific, USA), tooth surfaces were cleaned well with sterile cotton gauze to remove periodontal tissue. To expose the pulp and achieve decoronation of the teeth we used around the cement enamel junction a low speed hand piece and a sterile diamond stone with continuous sterile saline irrigation from a syringe to avoid overheating and death of pulp cells [4].

After washing with sterile PBS (Biowest, China), pulp tissue was sliced into small pieces (approximately 2 mm) [14]. Using the enzymatic digestion method, pulp tissue was processed in a solution of 0.1% collagenase type I (Lonza Verviers SPR, Belgium) for 1 h at 37 °C, filtered through a 70-μm cell strainer, double amounts of DMEM were used to stop the action of collagenase solution, and the tube was centrifuged at 1800 rpm for 10 min [15].

The cultured cells were then incubated at 37 °C in a humidified atmosphere containing 5% CO_2_ and the cultured cells were monitored using an inverted microscope (Olympus GX 53). The medium was changed every three days. Upon reaching 70–80% confluency of adherent primary cells, the cells were harvested using 0.25% trypsin/EDTA (Gibco, Grand Island, NY) and expanded to passage 4. The third and fourth passages were used in this study [16].

### Observation of cell morphology

DPSCs were morphologically evaluated under an inverted microscope to determine their number, viability, morphological changes, and confluency. DPSCs were identified based on their fibroblast-like morphology and colony-forming ability [17].

### The characterization of DPSCs was confirmed using flow cytometry

DPSCs were analyzed in the second passage (P2) to confirm their stem cell nature. An aliquot of 1 × 10 4 cells was evaluated by flow cytometry for surface markers of mesenchymal stem cell CD73 and negative staining for markers of hematopoietic stem cell CD45. The cells were incubated in staining buffer (PBS with 2% FBS and 0.1% sodium azide) containing anti-CD45 and anti-CD73 for 30 min on ice. Cells stained with the appropriate isotype-matched immunoglobulins were used as the negative controls. After staining, the cells were fixed with 2% w/v paraformaldehyde and analyzed using a NAVIOS flow cytometer [18].

### Preparation of odontogenic differentiation medium

Odontogenic induction medium (OM) was prepared by supplementing the growth medium with b-glycerophosphate, 0.2 mmol/L ascorbate-2-phosphated, and 100 nmol/L dexamethasone [19].

### Scaffolds preparation

Briefly, a solvent casting/particulate leaching technique was used for scaffold preparation. The scaffolds were composed of a mixture of polycaprolactone (PCL) polymer, nano-chitosan, and hydroxyapatite (and/or Mg-hydroxyapatite) prepared from eggshells at a ratio of 50:30:20 wt. First, a 10% (w/v) PCL solution was obtained by dissolving 6 g of PCL pellet in 60 ml of dry chloroform under vigorous mechanical stirring for approximately 2 h. The gradual addition of PCL pellets prevented sudden clumping if all pellets were added simultaneously. Nanochitosan (3.6 g) was then gradually added, followed by nano-HA and/or/ or 2.4 g Mg-hydroxyapatite. Sodium chloride (NaCl) crystals with particle size of 250-500 µm, was applied as porogens (pore-producing particles) at a PCL/NaCl ratio of 1:5. This porogen was slowly added to the PCL solution to obtain a homogeneous mixture. The composite mixture was directly poured into a 10 cm petri dish and dried. After complete evaporation of the solvent, the scaffold was detached from the Petri dish and directly immersed in a 200 mL clean beaker containing distilled water for 24 h. For the first 2 h, distilled water was exchanged every 30 min to extract NaCl crystals (porogen) from the scaffolds. For cell culture tests, scaffolds were subjected to ultraviolet radiation at a wavelength of 254 µm using a UV lamp for 15 min [20].

### Scanning electron microscopy of fabricated scaffolds

SEM (EM3200, KYKY, Beijing, China) examination of the synthesized scaffolds was performed to further investigate their characteristics of the prepared scaffolds, such as surface morphology and pore dimensions. In addition, the cells were seeded onto the scaffold to exclude the presence of cellular elements.

### X- ray diffraction analysis (XRD) characterizations of fabricated scaffolds

XRD, Pw 1390 channel control (manufactured by Philips, Holland) with CuK_α_ radiation, 40 kV and 25 mA was used to determine the chemical composition of the constant's phase's average crystal sizes the HAP and Mg-HAP powders. The specification criteria of XRD were adjusted at 2θ range = 5°–60° and λ = 1.54058 Å at a scanning speed of 2°/min. Each sample was used to fill the aluminum mold of the diffractometer with an average thickness of about 1.0 mm. The phases were identified by correlation with the corresponding Joint Committee on Powder Diffraction Standard Card (JCPDS).

The average crystallite size (D_*p*_) of the synthesized HAP powders was calculated from XRD using the Scherrer equation [21].

$${D}_{p} = \frac{0.94\lambda }{{\beta }_{1/2 }\mathrm{cos }\theta }$$

λ = the wavelength of the X-rayβ = the full width at half maximum (FWHM) of the peak at the maximum intensity.θ = the diffraction angle.

### Preparation of the bioactive materials

Under aseptic conditions, the three bioactive materials used in this study were prepared according to the manufacturer’s instructions with a disc width of 5 mm and thickness of 1 mm in a Teflon mold to ensure dimensional accuracy (Table 1). AB (Pulpdent USA) was mixed using a dispensing syringe, applied to a 1 mm-thick layer, and then cured for 5 s using a curing light device. TC (BISCO, USA) was dispensed from a syringe, applied in one layer of 1 mm depth, and then light-cured using an RTA light cure device (Woodpecker, China) for 20 s for 1 mm thickness layer [22].

**Table 1:** Bioactive materials used in this study

Material	Composition	Manufacturer
Activa bio active Base/Liner (AB)	Blend of diurethane and other methacrylates with modified polyacrylic acid (∼53.2%), silica (∼3.0%), and sodium fluoride (∼0.9%)	Pulpdent USA
TheraCal LC (TC)	Portland cement (30–50%), polyethelyene glycol dimethacrylate (10–30%), and barium zirconate (1–10%)	BISCO,USA
Mineral trioxide aggregate (MTA)	Portland cement, 4:1 bismuth oxide, calcium, silicon and Aluminum. liquid component is consisting H2O2	Angelus, Brazil

MTA (Angelus, Brazil) was mixed with a spatula in the shape of a disc with a diameter of 5 mm and thickness of 1 mm. The three bioactive materials were incubated in a CO_2_ incubator (Thermo Scientific, USA) at 37 °C and 100% humidity for 24 h to ensure complete setting, after which they were sterilized using ultraviolet light for 30 min [23].

### Preparation of material extracts

After incubation of the prepared materials, 1 mL of DMEM was added to each well and incubated at 37 °C and 95% relative humidity for 24 h. Aliquots were then extracted and treated every 3 days with this solution [24].

Study design and sample size calculation.

The sample size was calculated using (G* Power) computerized software guided by the results of a published study [25], yielding a minimum of 120 samples (10 samples/group). The sample size was increased to (12 per group) for teeth that may have been lost during the experiment (effect size = 0.40, Pooled SD = 1.48, alpha (α) = 0.05 and Power (β) = 0.95).

Randomization and blinding.

Each scaffold sample was randomly assigned to be treated with bioactive material extract. The scaffold samples were coded and divided into groups and subgroups, with each group placed in an opaque envelope. At the time of addition, the operator was aware of the type of bioactive materials used. A random sequence was generated using computer software (http://www.random.org/) [26].

The qRT-PCR laboratory data collector and SEM operator were blinded, and they were blinded to the bioactive material used to treat each group. Additionally, the data analyst who conducted the statistical analyses was blinded. Because bioactive materials must be exposed and cannot be masked, the operator cannot be blinded.

### Cell seeding on scaffolds and adding of bioactive materials

Forty-eight pieces of the PCL-NC-HA scaffold were placed in forty-eight sterile cell culture plates (Nunc, Germany), and the other forty-eight pieces of Mg-HA scaffolds were placed in forty-eight cell culture plates and primed in a biomimetic microenvironment for 2 h at 37 °C. The scaffolds were then covered with 100 μl μL complete culture medium containing DPSCs. These scaffolds were incubated for 2 h at 37 °C and 5% CO_2_ to provide initial cell attachment and then fully covered with previously prepared complete culture media. After 48 h. DPSCs were transferred to odontogenic differentiation medium (OM) and bioactive material extract aliquots previously prepared for all experimental samples, except for the positive control group, which only contained DPSCs and OM, and the negative control group, which contained DPSCs in complete media only. The media were changed every 3 d for two weeks duration [27].

### Grouping of samples

One hundred and eight samples were included in this study (*n* = 120). The positive control group (*n* = 12) only had DPSCs in the OM. The negative control group (*n* = 12) contained DPSCs in the culture media with no scaffold or OM.

**Fig. 1:**
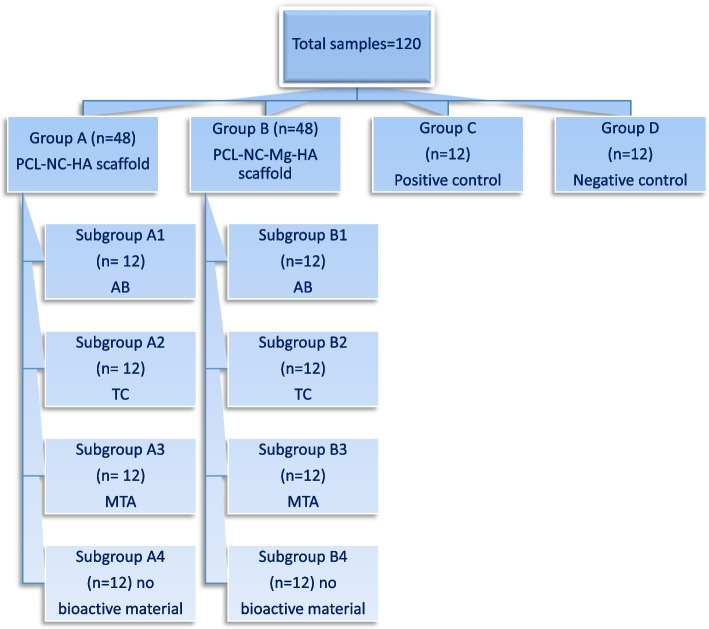
Flow chart described grouping of samples

The remaining 96 samples were divided into two main groups (*n* = 48) according to scaffold composition and further subdivided according to the added bioactive material, as shown in the flowchart (Fig. 1).

### Methods for evaluating the odontogenic differentiation potential of DPSCs

#### Cell morphology observation

DPSCs from all groups were morphologically estimated under an inverted microscope during the experiments to evaluate morphological changes in the cells and cell confluence.

Scanning Electron Microscopy (SEM) of the DPSCs-populated scaffold.

After two weeks, one sample from each group was rinsed with PBS and immersed in 2.5% glutaraldehyde for two hours. After three PBS rinses, samples were fixed with 1% osmium tetroxide for two hours. They were then rinsed thrice with PBS before being dehydrated with graded ethanol (30%-100%). Then, the samples were immersed for 15 min at each concentration. The solutions were then rinsed and the samples were dried in a hood. The samples were gold-coated and examined under SEM for viability, morphology, adhesion affinity, and matrix deposition [28].

### Gene expression analysis using real-time polymerase chain reaction

DSPP is a marker of early odontogenic differentiation that functions during primary dentinogenesis and is involved in odontoblastic differentiation [14]. The present study compared DSPP expression in all experimental samples to measure odontogenic differentiation of DPSCs, after which the cells were cultured for 14 days. RNA was extracted from each sample using the miRNeasy Mini Kit and converted into complementary DNA (cDNA) using reverse transcriptase. Glyceraldehyde-phosphate-dehydrogenase (GAPDH), designed by Macrogene (Korea), was used as the housekeeping gene [29].

Real-time polymerase chain reaction (qRT-PCR) (Applied Biosystems, USA) products were quantified using SYBR Master Mix in a real-time PCR system. After real-time PCR, the amplification efficiency of the genes was analyzed using the comparative cycle threshold (CT) method [30].

### Statistical analysis

The Shapiro–Wilk test was used to verify the normal distribution of the data. The data were parametric and normally distributed. The descriptive statistics of the fold-change of the tested gene DSPP included the mean, standard deviation, median, minimum, and maximum. An independent samples t-test was used to compare the fold-change in DSPP between the groups. One-way ANOVA was used to compare the fold-change in DSPP between subgroups. Tukey’s test was used for multiple comparisons of the fold-change in DSPP between the two subgroups. P was set than 0.05. Data were analyzed using (Statistical Package for the Social Sciences version 25).

## Results

### Stem cell characterization and identification

**Fig. 2:**
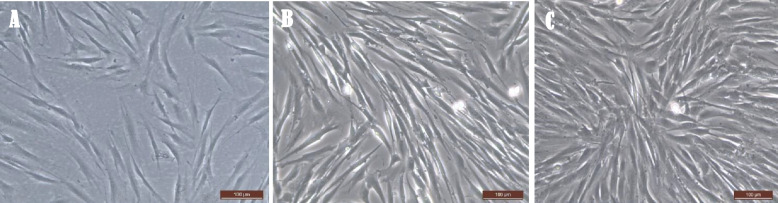
Phase contrasts of cultured DPSCs showing: **A** microphotograph of primary cell culture gained attachment and fibroblast-like morphology. **B** A microphotograph of primary cell culture of DPSCs exhibited 70% confluency after 5 days of seeding. **C** A microphotograph of DPSCs during the 2nd passage showing colonies and 80% confluency after 4 days of subculture. (X 100)

After 10 days, the primary cultures reached confluence. The morphology of the DPSCs cultures was fibroblast-like (elongated spindles) with abundant cytoplasm and large nuclei. Subcultures grew faster than primary cultures and reached confluence within half of the time (6-7d). The fibroblastic morphology of the cells and their tendency to form colonies were maintained throughout the culture period (Fig. 2).

### Flow cytometric analysis

**Fig. 3:**
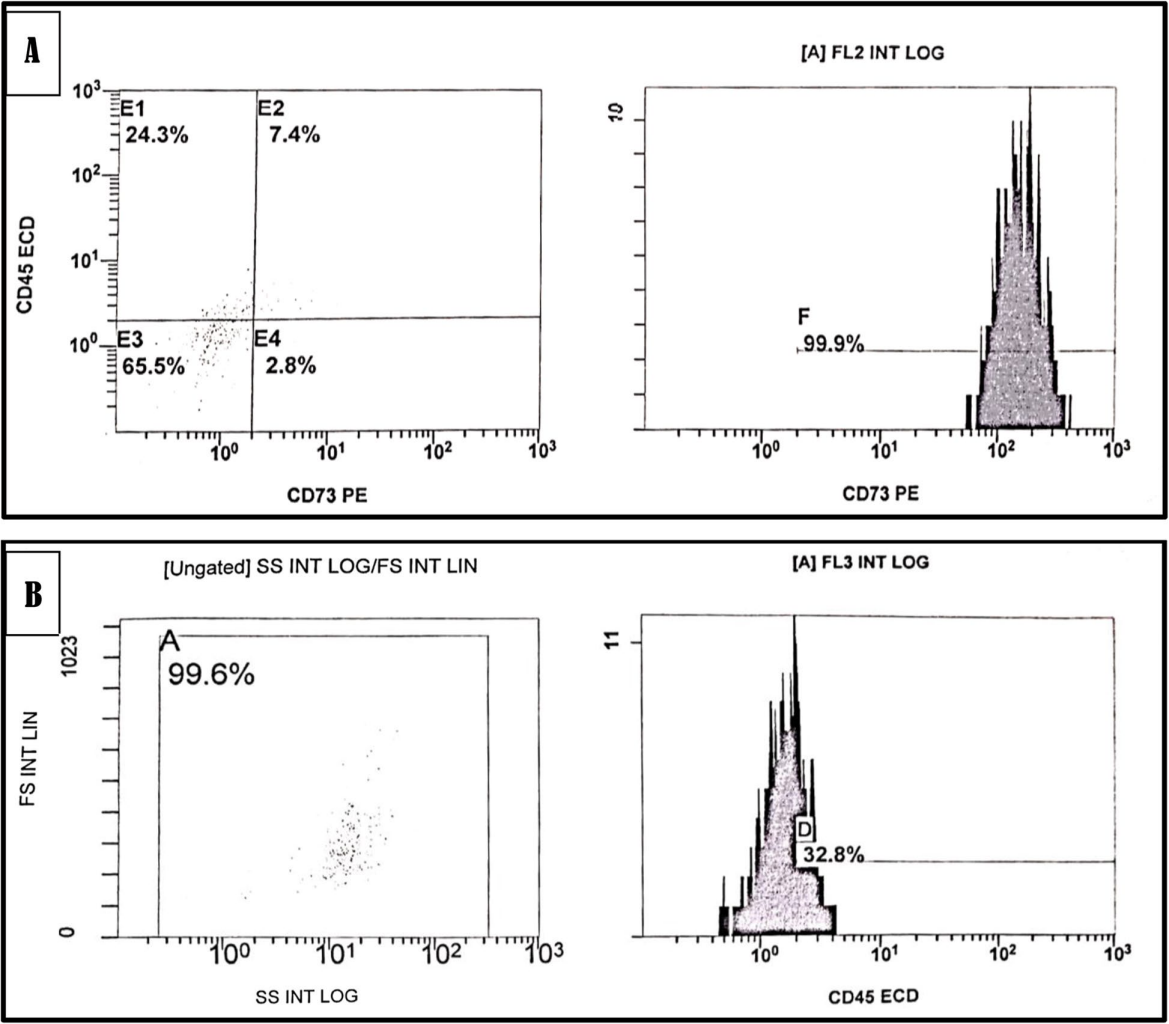
Photomicrographs showing flowcytometric analysis of CD73 **A**) CD45 **B**) antigens on DPSCs

At the end of the second passage, most cells showed positive expression of the mesenchymal stem cell marker (CD73) and negative expression of the hematopoietic cell marker (CD45). The percentage of positive cells was 99.9% for CD73 and 32.8% for CD45 (Fig. 3).

### SEM images of the prepared scaffolds

**Fig. 4:**
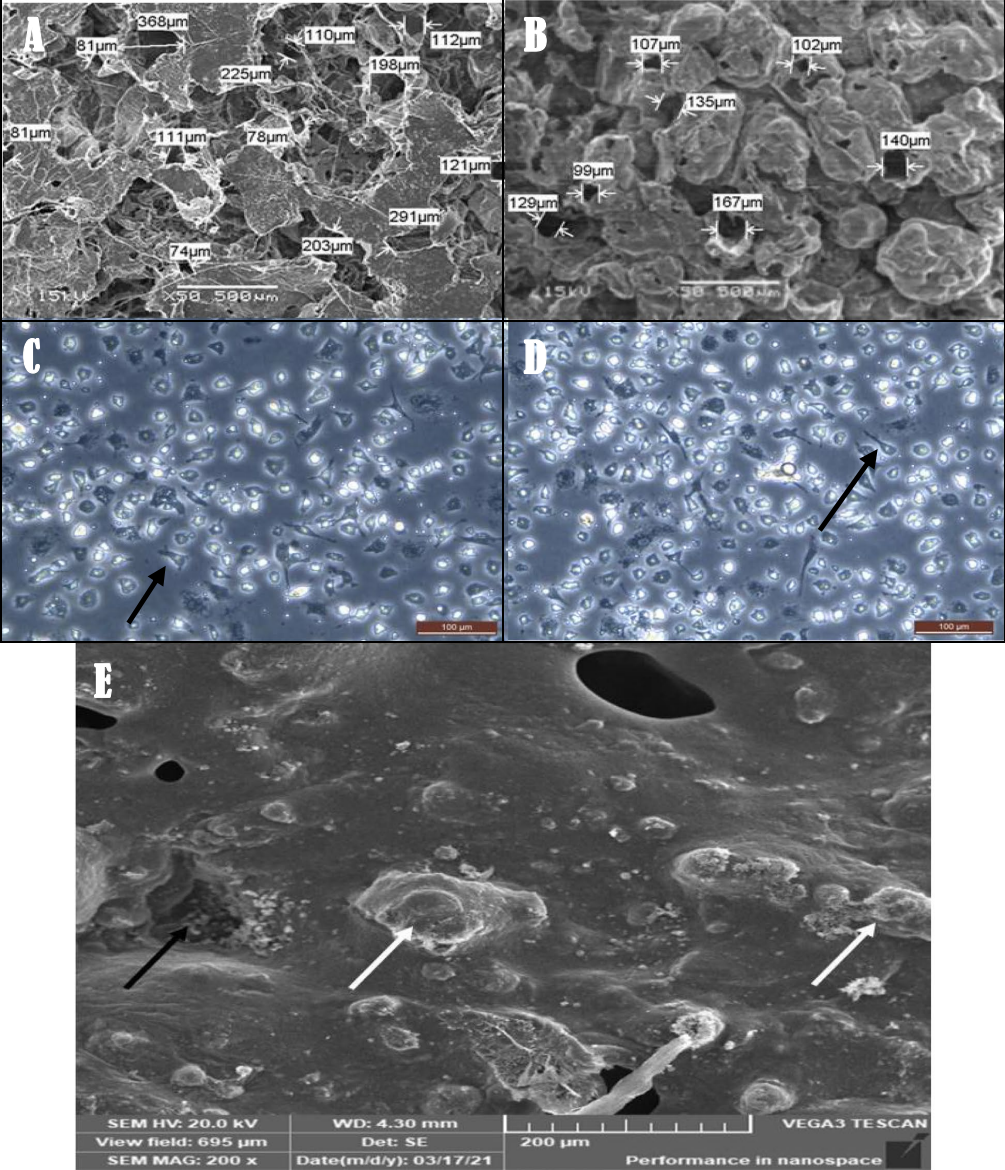
SEM microphotograph showing, **A** Average pore sizes (143 µm) of PCL-NC-HA scaffold. **B** Average pore sizes (126 µm) of PCL-NC-Mg-HA scaffold. Phase contrasts of DPSCs 14 days after odontogenic differentiation induction showing: **C** Transformation of the cultured DPSCs morphology after being induced using OM in the positive control group (black arrows) refers to the elongated cells. **D** Transformation of the cultured DPSCs morphology after odontogenic differentiation induction using OM in the positive control group from another sample confirmed the transformation that occurred in the treated cells. (100X). **E** Scan electron micrograph of scaffold from sample of subgroupA2 that treated with Theracal LC material extract showing cells attached in groups on the scaffold's surface properly (black arrows). Many mineralized nodules were formed on the scaffolds surface (white arrow)

Scanning electron micrographs of the surface morphology and pore dimensions of the synthesized empty scaffolds revealed that the scaffolds had a highly porous structure with well-defined interconnected open pores and average pore dimensions of 143 µm and 126 µm (average size), respectively (Fig. 4 A and B). The cells that were cultured on the scaffold and had physical contact with the biomaterials are illustrated in (Fig. 4 E).

### X-ray diffraction (XRD) of the scaffolds particles

**Fig. 5:**
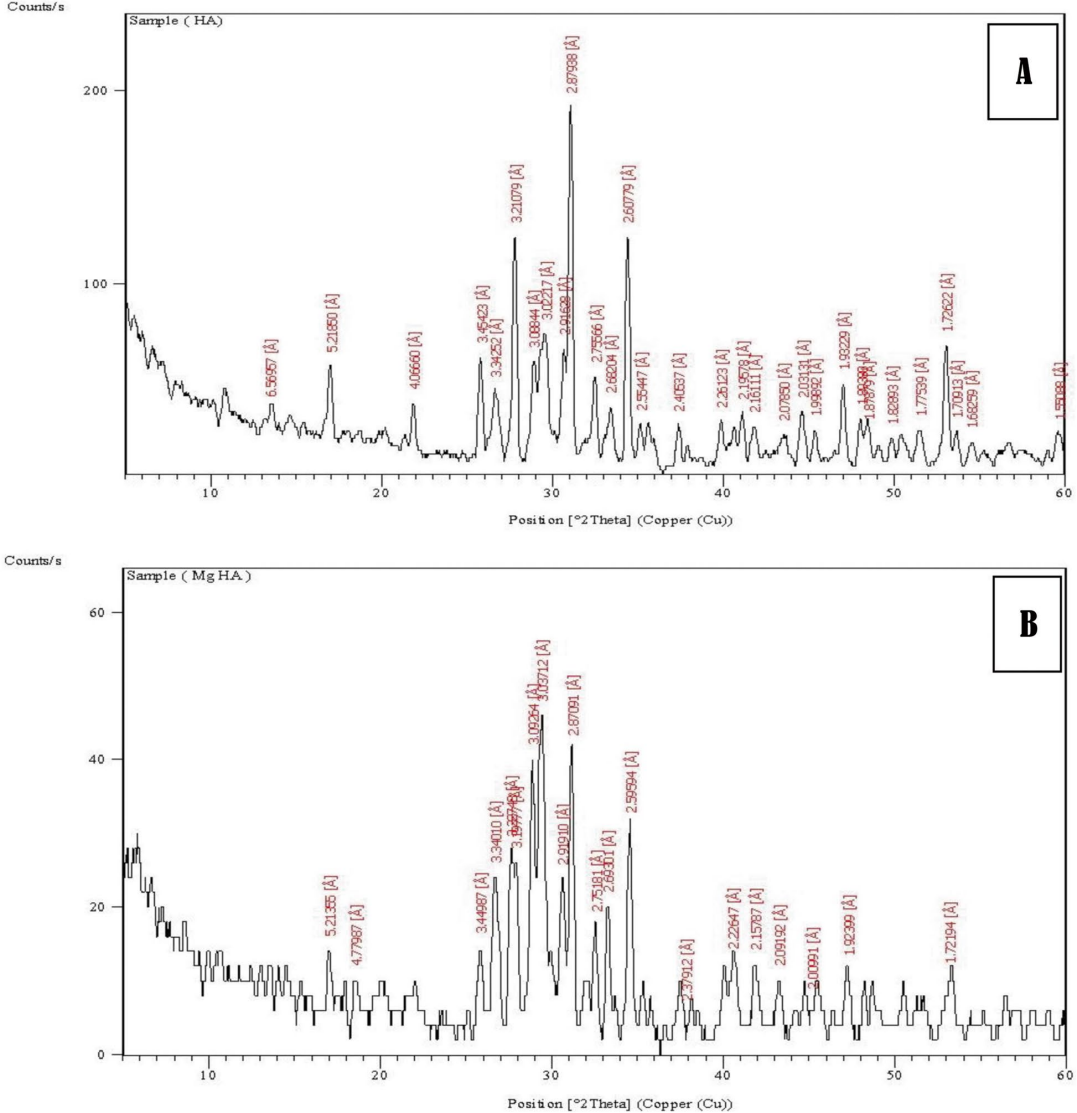
X-ray diffraction of synthsizic scaffold: **A** Showed HAP powder crystal size in range of 53 nm. **B** Showed Mg- HAP powder crystal size in range of 15 nm

X-ray diffraction pattern of the synthesized HAP and Mg-HAP powders in Fig. (32 and 33) revealed similar XRD pattern peaks of phase of hydroxyapatite and Mg- hydroxyapatite respectively. On the other hand, the samples showed the presence of minor amounts of beta-tricalium phosphate (*β*-TCP) as a secondary phase. The sample of HAP revealed crystallite size 53 nm, while the Mg- HAP sample showed the average crystal size in range of 15 nm (Fig. 5).

### Measurement of odontogenic differentiation potential of DPSCs after 14 days

#### Cell morphology after induction of odontogenic differentiation

After 14 days of DPSC induction, cells in the positive control group showed a great transformation in their morphology and loss of fibroblast-like appearance with shrinkage and shortening of their cytoplasmic processes; however, the differentiated odontoblast-like cells still exhibited a slightly elongated morphology (Fig. 4 C and D).

### SEM of cultured DPSCs

**Fig. 6:**
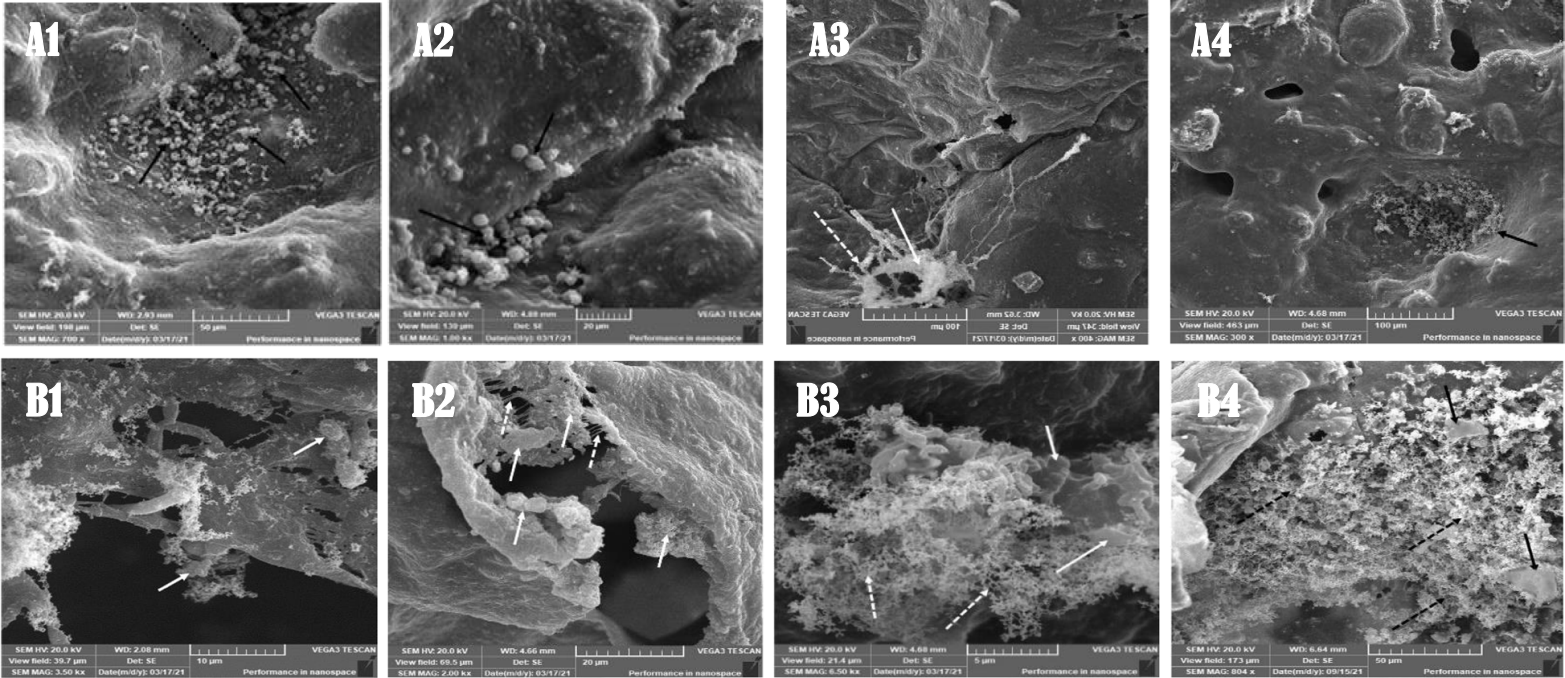
SEM micrographs of DPSCs on scaffold of different subgroups. **A1**) subgroup showing AB extract (black arrows), cells seeded and attached to the scaffold surface and some cells became elongated (dashed arrow). **A2**) subgroup showing cells attached on the scaffold's surface (black arrows). **A3**) subgroup showing cells attached, hanged on the scaffold's pores (arrows) and secreted foam-like matrix (dashed arrow). **A4**) subgroup showing cells attached and seeded on the scaffold's surface properly, cells also showing evidence of matrix formation. **B1**) subgroup showing cells attached and seeded on the scaffold's surface with matrix formation. **B2**) subgroup showing cells attached on the scaffold's surface (arrow) forming extracellular thread-like matrix (dashed arrow). **B3**) subgroup showing cells attached and expanded on the scaffold's surface (arrow) with the formation of sponge -like matrix in between the cells (dashed arrow). **B4**) subgroup showing cells expanded on the scaffold (arrow) and form a spongy like structure on the surface of the scaffold (dashed arrow).A) PCL-NC-HA scaffold, B) PCL-NC-HA scaffold, 1) Activa bioactive, 2) TheraCal, 3) MTA 4) Scaffold only no bioactive material

Evaluation of cell attachment and morphology by SEM microphotographs of samples from group A and B showed that the scaffolds supported the attachment and the growth of DPSCs inside their porous structure, the SEM microphotographs also revealed that there were mineralized nodules formed on the surface of the seeded scaffolds. The best result in terms of cell viability, growth, and attachment were observed in group B3 compared to the other groups, indicating odontoblastic transformation of the cultured DPSCs (Fig. 6).

### DSPP gene expression in the cultured differentiated cells

The results of the present study showed that all samples showed upregulation of the target gene DSPP compared with the negative control group. Group B showed a significantly higher fold-change in the target gene DSPP than group A in all subgroups. For group A, the highest mean fold change was recorded in subgroup A3, followed by subgroups A1 and A2, and the lowest mean fold change was noted in subgroup A4. There was no significant difference between the mean fold changes of subgroups A1 and A2, or between subgroups A2 and subgroupA4. Significant differences were observed among other subgroups. For group B, the highest mean fold change was recorded in subgroup B3, followed by subgroups B2 and B1, and the lowest mean fold change was noted in subgroup B4. There was no significant difference between the mean fold changes in subgroups B1 and B2. Significant differences were observed between the subgroups (Table 2).

**Table 2:** Comparison of fold change between subgroups for each group

Subgroup	X	SD
A1(Activa bioactive on PCL-NC-HA scaffold)	8.15a	.24
A2 ( TheraCal on PCL-NC-HA scaffold)	7.98a,b	.37
A3 (MTA on PCL-NC-HA scaffold)	8.74c	.31
A4 (PCL-NC-HA scaffold)	7.71b	.40
B1(Activa bioactive on PCL-NC-HA scaffold)	10.08 a	.36
B2 ( TheraCal on PCL-NC-Mg-HA scaffold)	10.29 a	.34
B3 (MTA on PCL-NC-Mg-HA scaffold)	11.65 b	.37
B4 (PCL-NC-Mg-HA scaffold)	9.59c	.34
One Way ANOVA ( *P* Value)	<.001*	

*X* Mean, *SD* Standard deviation

^*^p is significant at 5%, different letters in the same column indicate significant difference between each 2 subgroups, (Tukey, p.05)

## Discussion

Somatic and adult stem cells are undifferentiated cells found in the body after the development of differentiated cells. The function of these cells allows for healing, growth, and replacement of cells that are lost every day. DPSCs are a useful type of stem cell for tissue engineering because they are easy to access surgically, can be cryopreserved, and produce more dentin tissue than other types of stem cells. Numerous studies have shown that DPSCs can differentiate into odontoblast-like cells [31–33].

Previous studies [34, 35] examined the odontogenic differentiation of DPSCs into odontoblast-like cells when cultured and seeded on different types of scaffolds. To optimally utilize DPSCs, carrier materials that are structurally similar to the natural extracellular matrix (ECM) at the nanoscale can be easily modified for specific applications. High-performance scaffolds for hard tissue engineering, such as teeth, must have a porous three-dimensional structure that can provide sufficient space for cell migration, adhesion, and proliferation. In addition, it must have proper toughness to promote stem cell odontogenic differentiation. Polycaprolactone (PCL) has received considerable attention as a scaffold material for tissue engineering because of its good biocompatibility, low cost, and variable biodegradation characteristics. HA is a major inorganic component of hard tissues in the human body and can be used as a suitable material for improving cell proliferation and differentiation [36, 37]. Therefore, incorporating HA into polymer scaffolds such as PCL is a promising approach for preparing hard tissue scaffolds with proper mechanical strength. HA is an important inorganic component of the hard tissues in the human body and can be used to improve cell proliferation and differentiation. Therefore, incorporating HA into polymer scaffolds, such as PCL, is a promising approach for creating mechanically strong hard tissue scaffolds [38, 39].

Chitosan is a partially deacetylated derivative of chitin found in exoskeletons of arthropods. Its bioactive properties in addition to degradation properties in the body to non-harmful and nontoxic compounds were very attractive for the use in the dental tissue engineering field of study [40].

Therefore, a composite of HA and chitosan therefore is expected to be a good biomaterial for bone tissue engineering. Vagropoulou et al. [41]. In a recent study, the effect of a composite scaffold composed of HA combined with chitosan on DPSCs was investigated [13].

In the present study, we fabricated a composite scaffold that combined PCL, chitosan, and HA to investigate the combined effects of these three scaffold materials on DPSCs. In addition, the results revealed acceleration of cellular adhesion, enhancement of odontogenic differentiation, and cell proliferation. Furthermore, we used another type of scaffold by substitution Mg^+2^ in HA. Mg-based HA scaffolds have already been introduced as promising materials in the tissue engineering of mineralized tissues because the continuous release of Mg^+2^ ions during the gradual dissolution of the scaffold may provide the necessary chemical stimulus for stem cell proliferation and odontogenic differentiation [42].

Jun et al. [9] investigated some bioactive materials regarding their odontogenic differentiation abilities, they Activa bioactive showed less odontogenic differentiation effect when compared with TC. identified early genetic changes related to odontogenic differentiation when MTA is applied to DPSCs, and stated that the exposure of human dental pulp stem cells DPSCs to MTA increases the transcripts of dentin sialophosphoprotein DSPP gene, and the expression of the gene DSPP increases in the odontogenic differentiation of DPSCs, suggesting the odontogenic differentiation ability of the bioactive material MTA on DPSCs [10].

Regarding the bioactive material in the current study, we believe that the most convenient method is the material extraction method, instead of direct contact between the bioactive materials and DPSCs, to avoid crushing or abrasion of the DPSCs seeded in the scaffolds, which is in agreement with the results of Hengameh et al. [24] and Jun et al. [9].

To measure the odontogenic differentiation of DPSCs we used gene expression analysis by quantitative real time PCR method as the most precise method to measure the odontogenic differentiation of the DPSCs [39]. The gene of choice in the present study, the expression of DSPP, which is primarily expressed in odontoblasts, was higher during primary dentinogenesis, suggesting a more aggressive role of DSPP in odontoblast differentiation and function during primary dentinogenesis [43].

The results of the present study showed that all samples exhibited upregulation of the target gene, DSPP. Group B samples, which contained a scaffold composed of polycaprolactone, nano chitosan, and Mg-substituted HA, showed a significantly higher fold change of the target gene expression when compared with the other samples seeded on the other type of scaffold that did not contain Mg, strongly supporting the higher odontogenic differentiation effect of Mg HA polycaprolactone-nano-chitosan on DPSCs than the other types of scaffolds that did not contain Mg, which is in agreement with many other previous studies, such as Theocharidou et al. [27] and Qu et al. [44].

The results also revealed that the highest fold change in DSPP was found in subgroupA3 and B3, which were treated with MTA, which is in agreement with the results of Saberi et al. [45]. The lowest fold change was found in subgroups A4 and B4, which were not induced by the bioactive materials. There was no significant difference between subgroups A1and A2 or between B1and B2 which were induced by AB and TC, respectively, which is in disagreement with the results of Jun et al. [9]. This could be due to another factor added to this study: the combined effect of the scaffolds and bioactive materials.

The suggested hypothesis was rejected, where the MTA and PCL-NC-Mg-HA scaffolds had a better odontogenic differentiation effect on cultured DPSCs.

## Conclusion

Within the limitations of the present study, the PCL-NC-Mg-HA scaffold had a better odontogenic differentiation effect on the cultured DPSCs, and the combination of this scaffold and the bioactive materials positively affected the induction of DPSCs into odontoblast-like cells. MTA is the best bioactive material for inducing odontogenic differentiation of cultured DPSCs compared to AB and TC.

Further preclinical studies are needed to advance clinical applications, necessitating additional testing to demonstrate their safety and efficacy However, additional studies are necessary to clarify its mechanism of action. Furthermore, it was discovered that the same material had different effects on the fate of stem cells from different sources, and other tests are required to characterize the scaffolds.

## Data Availability

The datasets generated and analyzed during the current study are not publicly available due to (ownership of data) but are available from the corresponding author on reasonable request.

## Abbreviations

DPSCs: Dental pulp stem cells
AB: Activa bioactive (base/liner)
TC: TheraCal LC
MTA: Mineral trioxide aggregate
PCL-NC-HA: Polycaprolactone-nano-chitosan + synthetic hydroxyapatite
PCL-NC-Mg-HA: Polycaprolactone-nano-chitosan + synthetic Mg-substituted hydroxyapatite
DSPP: Dentin Sialophosphoprotein
qRT-PCR: Quantitative real-time polymerase chain reaction
Mg: Magnesium
OM: Odontogenic differentiation medium
DMEM: Dulbecco's modified Eagle's medium
P1: Frist passage
P2: Second passage
P3: Third passage
P4: Fourth passage
